# Chemical Constituents of *Muehlenbeckia tamnifolia* (Kunth) Meisn (Polygonaceae) and Its In Vitro α-Amilase and α-Glucosidase Inhibitory Activities

**DOI:** 10.3390/molecules21111461

**Published:** 2016-11-02

**Authors:** María Torres-Naranjo, Alirica Suárez, Gianluca Gilardoni, Luis Cartuche, Paola Flores, Vladimir Morocho

**Affiliations:** 1 Departamento de Química, Universidad Técnica Particular de Loja, Loja 1101608, Ecuador; mbeltorres15@hotmail.com (M.T.-N.); gianluca.gilardoni@gmail.com (G.G.); lecartuche@utpl.edu.ec (L.C.); pkflores@utpl.edu.ec (P.F.); 2 Facultad de Farmacia, Universidad Central de Venezuela, Caracas 1040, Venezuela; alirica1@yahoo.es; 3 Prometeo Project Researcher, SENESCYT, Quito 170516, Ecuador

**Keywords:** *Muehlenbeckia tamnifolia*, α-glucosidase inhibition, α-amilase inhibition, linoleic acid

## Abstract

The phytochemical investigation of *Muehlenbeckia tamnifolia*, collected in Loja-Ecuador, led to the isolation of nine known compounds identified as: lupeol acetate (**1**); *cis*-*p*-coumaric acid (**2**); lupeol (**3**); β-sitosterol (**4**) *trans*-*p*-coumaric acid (**5**); linoleic acid (**6**) (+)-catechin (**7**); afzelin (**8**) and quercitrin (**9**). The structures of the isolated compounds were determined based on analysis of NMR and MS data, as well as comparison with the literature. The hypoglycemic activity of crude extracts and isolated compounds was assessed by the ability to inhibit α-amylase and α-glucosidase enzymes. The hexane extract showed weak inhibitory activity on α-amylase, with an IC_50_ value of 625 µg·mL^−1^, while the other extracts and isolated compounds were inactive at the maximum dose tested. The results on α-glucosidase showed more favorable effects; the hexanic and methanolic extracts exhibited a strong inhibitory activity with IC_50_ values of 48.22 µg·mL^−1^ and 19.22 µg·mL^−1^, respectively. Four of the nine isolated compounds exhibited strong inhibitory activity with IC_50_ values below 8 µM, much higher than acarbose (377 uM). Linoleic acid was the most potent compound (IC_50_ = 0.42 µM) followed by afzelin, (+)-catechin and quercitrin.

## 1. Introduction

Diabetes is defined as a complex metabolic disorder that affects protein, carbohydrate and fat metabolism, all due to insulin insufficiency or insulin dysfunction. According to the World Health Organization (WHO), this disease affects more than 382 million people around the world [1]. Hyperglycemia and dyslipidemia factors, increased in diabetes, are involved in the cardiovascular failures that frequently affect patients. Diabetes is considered a public health problem, affecting people in both developed and developing countries, with no difference of race. According to the diabetes country profiles, diabetes mellitus accounts for 4% of total deaths in Ecuador, and ranks sixth in the population’s death causes, followed by respiratory diseases [2]. For this reason, it is a priority issue for public health that needs the attention of both the government and academy. Folk medicine is well recognized in Ecuador, where almost 80% of the population uses plants as their primary source for medical treatment. Several plants are successfully used as antidiabetics in traditional medicine, and the WHO has recommended the assessment of these plants and their product derivatives for their potential use as oral therapy [3]. A scientific validation of medicinal plants is necessary in order to prove efficiency, potency and security. Although many plants have beneficial effects, they can also be toxic, especially in long treatments such as those required in antidiabetic therapies. One of the therapeutic approaches used to treat diabetes consists of the inhibition of enzymes, which can hydrolyze the polysaccharides and convert them into simple sugars. This type of enzyme, such as alpha-amylase, catalyzes the hydrolysis of the α-1-4-d-glycosidic linkage of glucose polymers such as starch. Αlpha-glucosidase acts by hydrolyzing disaccharides to glucose. Inhibitors of these enzymes can interrupt the absorption of glucose in the digestive tract, prolonging the carbohydrate digestion time and decreasing the rate of glucose absorption. As a consequence, the postprandial blood glucose level is lowered [3]. For this reason, both alpha-amylase and alpha-glucosidase inhibitory assays have been extensively used to test drugs and plant extracts.

Polygonaceae is a family characterized for the production of a large variety of secondary metabolites, many of which exhibit potential pharmacological activity, such as anthraquinones, naphthalenes, stilbenoids, steroids, glycosylated flavonoids, leucoanthocyanidins and phenolic acids, among others [4]. Some species have been reported to have antidiabetic potential, such as *Muehlenbeckia sagittifolia* [5], *Coccoloba uvifera* [6], *Rumex* spp. [4] and *Rheum* spp. [7,8,9]. In Ecuador, this family accounts for nine genera and 42 species [10]. The genus *Muehlenbeckia* is restricted to South America, Australia and New Zealand and includes ornamental and medicinal species. These species are frequently used to treat gastric ulcers, and are used as macerated leaves to treat kidney ailments and as infusions to relieve the pain of arthritis [11].

Phytochemical studies for species belonging to this genus have been reported. Glycosylated flavonoids such as catechin and quercitrin, isolated from *M. platyclada*, have shown antinociceptive and anti-inflammatory activities. A series of anthraquinones such as emodin, physcion and emodin-8-β-d-idopyranoside, isolated from the leaves of *M. hastulata*, showed promising antioxidant activity measured by the DPPH method [12,13].

*Muehlenbeckia tamnifolia* (Kunth) Meisn, known as *Anku yuyu lutu yuyu*, is used by indigenous communities in Ecuador to treat kidney diseases, in baths to relieve bone pain, as mouthwash for toothache and, in combination with other plants, to treat bumps and inflammation. It is also applied as a disinfectant and to treat purulent skin wounds [14]. Previous chemical studies on the roots of *M. tamnifolia* showed the presence of anthraquinones such as chrysophanic acid and emodin [15]. The aim of this work was to evaluate the inhibitory activities of different extracts and isolated compounds of *M. tamnifolia* on α-amylase and α-glucosidase.

## 2. Results and Discussion

**Figure 1:**
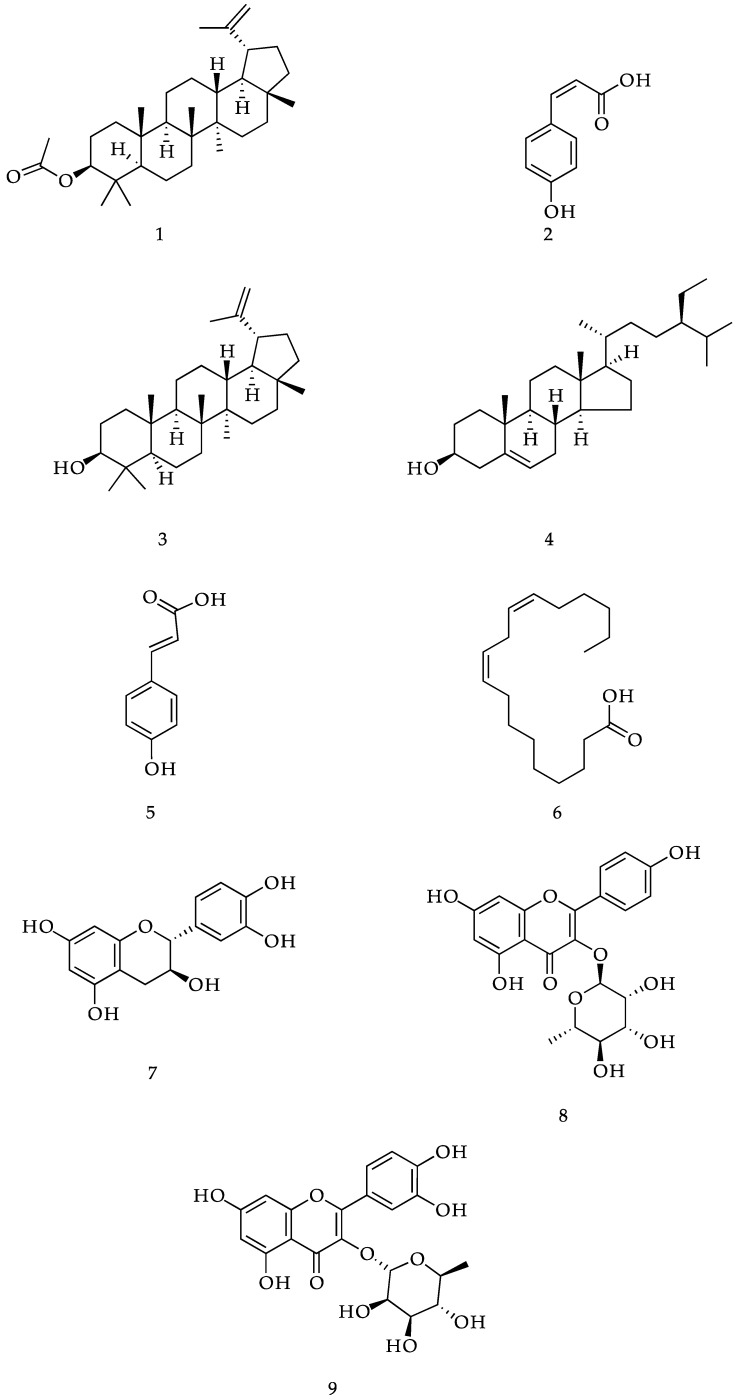
Structure of compounds (**1**–**9**) isolated from *Muehlenbeckia tamnifolia*.

The phytochemical study of *M. tamnifolia* led to the isolation and identification of nine compounds, whose structures are shown in Figure 1. The spectral properties of these known compounds, including ^1^H-NMR and ^13^C-NMR data, were identical to those previously described in the literature. Among the species of this genus, (+)-catechin (**7**) and quercitrin (**9**) have been reported only in *M. platyclada* [16]. Lupeol acetate (**1**), *cis*-*p*-coumaric acid (**2**), lupeol (**3**), β-sitosterol (**4**), *trans*-*p*-coumaric acid (**5**), linoleic acid (**6**) and afzelin (**8**) are reported here for the first time in this genus.

The ^1^H-NMR spectrum of compound **1** shows the presence of an acetyl group as a singlet at 2.05 ppm, typical of the methyl bonded to a carboxyl group. The ^13^C-NMR shows a signal at 173.8 ppm, consistent with the carbon atom of an ester group, and a signal at 21.4 ppm corresponding to the methyl group of an acetyl derivative. The remaining ^1^H- and ^13^C-NMR signals are similar to those obtained for compound **3** [17].

The ^1^H-NMR spectrum of compound **2** (Figure 1) displayed two doublets, one at 7.63 (2H, d, *J* = 8.8 Hz) and 6.79 (2H, d, *J* = 8.8 Hz) ppm, typical of a 1,4-substituted aromatic ring. Two doublets at 6.83 (1H, *J* = 12.5 Hz) and 5.82 (1H, *J* = 12.5 Hz) ppm revealed the presence of a *cis* olefin coupling, corresponding to H-7 and H-8 in the proposed structure. The above spectral features are in close agreement to those observed for *cis*-*p*-coumaric acid [18].

The ^1^H-NMR spectrum of compound **3** shows six singlets, corresponding to tertiary methyl groups, between 0.75 and 1.02 ppm and a singlet at 1.25 ppm, typical of a methyl group in an isopropenyl system. Two olefinic protons at 4.68 and 4.56 ppm are consistent with the methylene group of the same propylenic system. The ^13^C-NMR data shows the characteristic signals of C-3 at 79.2 ppm, C-20 at 151.1 ppm and C-29 at 109.5 ppm [17].

The ^1^H-NMR spectrum of compound **4** shows two signals, two methine groups at 3.51 and another at 5.35 ppm, consistent with H-3 and H-6 of a steroid nucleus. The ^13^C-NMR spectrum showed 29 carbon atoms including an oxymethine carbon signal at 72.0 ppm, two olefinic carbons at 140.9 and 121.9 ppm and two methylene carbon signals at 34.1 and 26.2 ppm, corresponding to C-23 and C-22 [19].

The ^1^H-NMR spectrum acquired for compound **5** (Figure 1) was almost identical to that of *cis*-*p*-coumaric acid (**2**), suggesting a close structural similarity between these two compounds. The difference is found in two doublets (*J* = 16.0 Hz) centered at 7.64 and 6.29 ppm which suggest the *trans-*derivative of compound **2** [20].

Compound **6** was characterized using GC-MS. Peak identification was carried out by comparing the recorded mass spectra with those present in the instrument library (WILEY 7n.l).

The ^1^H-NMR spectrum of compound **7** shows three signals corresponding to aromatic protons at 6.72, 6.76 and 6.83, corresponding to a 1,3,4-trisubstituted ring. The spectrum also shows the signals of two aromatic proton signals at 5.85 and 5.93 ppm, typical of a 1,2,3,5-tetrasubstituted ring. Two oxygenated methine protons signals are located at 3.97 and 4.56 ppm, consistent with H-3 and H-2 in the structure of catechin, respectively. Moreover, two methylenic diastereotopic proton signals can be observed at 2.55 and 2.87 ppm, which is consistent with position 4 of catechin. The ^13^C-NMR spectrum and DEPT experiment show five oxygenated quaternary aromatic signals at 157.8, 157.6, 156.9, 146.2 and 146.2 ppm, two non-oxygenated quaternary aromatic signals at 132.2 and 100.8 ppm, and five CH aromatic signals at 120.0, 116.1, 115.2, 96.3 and 95.5 ppm. Two oxygenated methine carbons signals at 82.8 and 68.8 ppm and one methylene carbon signal at 28.5 ppm are also present. The metabolite has been identified as the dextrorotatory enantiomer by polarimetry [21].

The ^1^H-NMR spectrum of compound **8** shows the proton signals of a para-disubstituted benzene ring at 7.77 and 6.93 ppm, those of a 1,2,3,5-tetrasubstituted benzene ring at 6.38 and 6.20 ppm, that of a typical anomeric proton doublet at 5.38 ppm and the signals of a sugar moiety between 4.20 and 2.00 ppm. Furthermore, the characteristic doublet of the rhamnose methyl group is present at 0.92 ppm. The ^13^C-NMR spectrum showed 21 signals, including the one of an α,β-unsaturated ketone at 179.6 ppm. Six signals of quaternary aromatic and olefinic oxygenated carbon atoms are present at 165.9, 163.2, 161.6, 159.3, 158.6 and 136.1 ppm, while two quaternary aromatic non-oxygenated atoms correspond to signals at 122.6 and 105.73 ppm. Other CH aromatic carbon atoms are represented by signals at 131.9 (positions 2′ and 6′), 116.5 (positions 3′ and 5′), 100.1 and 94.9 ppm. The rhamnose moiety anomeric carbon atom corresponds to the signal at 103.5 ppm [22,23].

The ^1^H-NMR spectrum of compound **9** shows the proton signals of a 1,3,4-trisubstituted benzene ring at 7.33, 7.31 and 6.91 ppm, and the remaining signals are in complete agreement with those previously reported for compound **8** [22].

### α-Amylase and α-Glucosidase Inhibition Activity

In the α-amylase assay only the hexanic extract was active at the maximum dose tested (625 μg/mL with a sample solution of 10 mg/mL). The other extracts and compounds exhibited inhibition percentages less than 35% at the tested conditions and were not included in this report.

For the α-glucosidase assay, both hexanic and methanolic extracts exhibited strong inhibitory activity and only four compounds were active. Three of them belonging to the flavonoid family and one belonging to the essential fatty acids group exhibited strong inhibitory activity, with IC_50_ values below than 8 μM (Table 1). Their inhibitory activities were even 48.5- to 754-fold higher than the commercial drug Acarbose (IC_50_ = 377 μM). The remaining five compounds exhibited no inhibitory activity over α-glucosidase at the maximum dose tested (less than 1% of enzyme inhibition) and only *trans*-*p*-coumaric acid exhibited 27% of enzyme inhibition. The activity of Afzelin (Kaempferol 3-*O*-rhamnoside) was 20-fold higher compared to the activity reported by Ajish et al. [24], who obtained an IC_50_ value of 81.16 μM. The method described previously in the methodology measures the pNP released during 60 min which allows us to obtain more reliable data by measuring enzymatic catalysis for a long period of time instead of short periods or even end point measurements. Additionally, using a high amount of enzyme can greatly affect the IC_50_ value obtained.

Moreover, the flavonoids have demonstrated to be moderate to strong inhibitors of α-glucosidase, with IC_50_ values below 100 μM [24,25,26,27,28]. Linoleic acid was demonstrated to be the most potent compound, followed by afzelin, (+)-catechin and quercitrin. PUFA (polyunsatured fatty acids) have been demonstrated to be good inhibitors of glucosidase, exhibiting different types of inhibition and potency, as demonstrated by Liu et al. [29], where C18 unsaturated fatty acids where more active versus longer fatty acid chains with more double bonds (or even no double bonds). The α-glucosidase inhibitors acting toward enzymes in the intestine have been shown to effectively delay the blood glucose level, lowering glucose absorption.

## 3. Materials and Methods

### 3.1. General Information

The NMR data were gathered by using a Varian Agilent (Walnut Creek, CA, USA, 400 MHz for ^1^H- and 100 MHz for ^13^C) in CDCl_3_ and CD_3_OD. Chemical shifts were reported in units (ppm) relative to the signal of Tetramethylsilane (TMS) and coupling constants (*J*) in Hz.

The GC-MS analyses were performed on an Agilent Technologies (Wilmington, DE, USA) 6890N gas chromatograph coupled to a mass spectrometer detector Agilent Technologies 5973N. The instrument was equipped whit a DB5-MS Agilent 122-5532 column (length 30 m, internal diameter 0.25 mm thickness of the stationary phase: 0.25 μm). Helium (1 mL/min) was the carrier gas and the injector was operated in split mode (ratio 1:1) at a temperature of 250 °C. The oven was programmed with an initial temperature of 50 °C for 1 min, then increased to 270 °C at 10°/min and kept at 270° for 25 min.

Silica gel 60 (Merck KGaA, Darmstadt, Germany, from 0.063 to 0.200 mm) and RP-18 (Merck 40–63 µm) were used as stationary phases for column chromatography. All organic solvents were bought in Brenntag (Brengtan, Guayaquil, Ecuador), then distilled. Optical rotations were measured using an Automatic Polarimeter (Jinan Hanon Instruments Co. Ltd. Jinan, China) MRC P810 with Hanon was used.

The α-amylase and α-glucosidase assays were evaluated using α-amylase from porcine pancreas (Type IV-B, Sigma A3176, St. Louis, MO, USA) and α-glucosidase from *Saccharomyces cerevisiae* (Tipe I, Sigma G5003, St. Louis, MO, USA). Starch was used as substrate for α-amylase and *p*-Nitrophenyl-α-d-glucopiranoside (*p-*NPG-Sigma, N1377) was used as substrate for α-glucosidase. For both enzymatic tests, a microplate reader (EPOCH 2, Biotek Instruments Inc. Winoosky, VT, USA) was used.

### 3.2. Plant Material

The leaves of *M. tamnifolia* were collected in the flowering stage in the locality of “Cerro Gañil”, province of Loja, Ecuador, in February 2015, at 2987 m.a.s.l. (Meters above sea level, coordinates 686617 N; 9602180 E). The plant material was identified by Nixon Cumbicus and, voucher specimens (PPN-PL-006) are deposited at the Herbarium HUTPL at the Universidad Técnica Particular de Loja.

### 3.3. Extraction and Isolation of Compounds

The dried leaves of *M. tamnifolia*, (500 g) were macerated for 1 h in a series of solvents according to a rule of increasing polarity: hexane (Hex), ethyl acetate (EtOAc) and methanol (MeOH), each solvent 6 L three times. The obtained solutions were filtered and concentrated under reduced pressure, to get three total extracts: 6.25 g (Hex) 6.35 g (EtOAc), and 63.34 g (MeOH).

The hexane extract (3 g) was submitted to silica column chromatography, with an extract/silica ratio of 1:55. The column was eluted according to a gradient of increasing polarity, from hexane-ethyl acetate 90:10 to 100% ethyl acetate, obtaining a total of 16 fractions (MM01-5/MM16-5).

The fraction MM01-5 was purified by column chromatography, with an isocratic system of Hex–CH_2_Cl_2_ 80:20 to get compound **1** (70 mg).

The fraction MM03-5 was purified by column chromatography, with an isocratic system of Hex–CH_2_Cl_2_ 90:10 to obtain compound **2** (10 mg).

The fraction MM04-5, eluted in isocratic conditions with Hex–EtOAc 80:20, afforded compound **3** (45 mg).

The fraction MM06-5, eluted in isocratic conditions with Hex–EtOAc 70:30, afforded compound **4** (20 mg).

The ethyl acetate extract (3 g) was fractionated by silica gel column chromatography, with an extract/silica ratio of 1:50 and an increasing polarity gradient of Hex–EtOAc from 95:5 to 100% ethyl acetate. Finally, the column was extracted with acetone 100%, obtaining a total of 20 fractions (MM01-12–MM20-12).

The fraction MM06-12 was purified by column chromatography, with an isocratic system of Hex–EtOAc 90:10, to obtain compound **5** (16 mg).

The fraction MM09-12 was eluted with an isocratic system of Hex–EtOAc 70:30 to get compound **6** (34 mg).

The methanol extract (5 g) was submitted to a partition with a mixture of MeOH–H_2_O 95:5 and, *n*-Hexane (1:1 volume ratio), obtaining a polar extract of 3.68 g. The polar extract was fractionated by silica gel column chromatography, eluting with increasing polarity from CH_2_Cl_2_–MeOH 95:5 to CH_2_Cl_2_–MeOH 70:30, obtaining a total of five fractions (MM01-40 to MM05-40).

The fraction MM02-40, was purified by direct phase column chromatography, eluting with increasing polarity gradient from EtOAc–Hex 80:20 to 100% EtOAc, to get compound **7** (13 mg) and compound **8** (13 mg).

The fraction MM04-40 was purified on reversed phase column chromatography, with an isocratic system of MeOH–H_2_O 90:10, to obtain compound **9** (15 mg).

### 3.4. Measurement of α-Amylase Inhibitory Activity

The inhibitory activity is measured according to the method reported by Xiao et al. [30] with slight modifications. Acarbose was used as the positive control. Starch solution was prepared by dissolving 1 g in 50 mL of 0.4 M NaOH and heated for 5 min at 100 °C. After cooling in iced water, the solution pH was adjusted to 7 with 2 M HCl and H_2_O was added to complete 100 mL. Sample solutions were prepared by dissolving 10 mg in 1 mL MeOH:H_2_O (50:50). Several dilutions in PBS (SIGMA-P4417) were made in case of getting complete enzyme inhibition. PBS solution (35 μL), substrate (35 μL) and sample (5 μL) solutions were mixed in a 96-well microtiter plate, and the mixture was pre-incubated at 37 °C for 1 min. Then, 20 μL of a 50 μg/mL α-amylase solution was added to each well. The plate was incubated for 15 min. The reaction was terminated by addition of 50 μL of 0.1 M HCl and then 150 μL of 0.5 mM iodine solution (0.5 mM I_2_ and 0.5 mM KI) were added. The absorbance was measured in a microplate reader (EPOCH 2, Biotek Instruments Inc. Winoosky, VT, USA) at 580 nm. Inhibitory activity (%) was calculated according to the formula described by Kusano et al. [31] as follows:

(1) Inhibition (%)=[1−(ABS2−ABS1)/(ABS4−ABS3)×100]

where *ABS*1 is the absorbance of the incubated solution containing sample, starch and amylase, *ABS*2 is the absorbance of incubated solution containing sample and starch, *ABS*3 is the absorbance of incubated solution containing starch and amylase, and *ABS*4 is the absorbance of incubated solution containing starch. IC_50_ value was calculated by curve fitting of data (GraphPad Prism 5.0, GraphPad Software, Inc., La Jolla, CA, USA). Acarbose was used as positive control.

### 3.5. α-Glucosidase Inhibition Assay

α-Glucosidase inhibitory activity was determined using a 96-well microtiter plate with *p*-nitrophenyl-α-d-glucopyranoside as the substrate, according to the method describe by Tao et al. [32], with slight modifications. Sample solutions were prepared by dissolving 10 mg in 1 mL MeOH:H_2_O (50:50). Several dilutions in PBS were made in case of getting complete enzyme inhibition. First, 75 μL of PBS (SIGMA-P4417) was mixed with 5 μL of the sample and 20 μL of the enzyme solution (0.15 U/mL in PBS pH 7.4), then it was pre-incubated at 37 °C for 5 min prior to the initiation of the reaction by adding the substrate. After pre-incubation, 20 μL of PNPG (5 mM in phosphate buffer, pH 7.4) was added and then incubated at 37 °C. The amount of *p*-nitrophenol (*p*-NP) released was measured in an EPOCH 2 (BIOTEK^®^) microplate reader at 405 nm for 60 min, recording the absorbance every 5 min. The results were expressed as inhibition percentage by means of the formula described by Choi et al. [33] as follows:

(2) Inhibition (%)=[(Ao−As)/Ao]×100

where *A*_0_ is the absorbance recorded for the enzymatic activity without inhibitor (control), and *A_s_* is the absorbance recorded for the enzymatic activity in presence of the inhibitor (sample). IC_50_ value was calculated by curve fitting of data (GraphPad Prism 5.0). Acarbose was used as positive control.

### 3.6. Physical and Spectral Data of Isolated Compounds

*Lupeol acetate* (**1**). C_32_H_52_O_2_ crystals (70 mg) ^1^H-NMR (CDCl_3_, 400 MHz): δ (ppm); 4.68 (1H, s, H-29b), 4.56 (1H, s, H-29a), 4.47 (1H, dd, *J* = 5.2, 10.8 Hz, H-3), 2.05 (3H, s, H-2), 1.68 (3H, s, H-30), 1.02 (3H, s, H-25), 0.94 (3H, s, H-28), 0.87 (3H, s, H-23), 0.85 (3H, s, H-24), 0.83 (3H, s, H-26), 0.78 (3H, s, H-27). ^13^C-NMR (CDCl_3_, 100 MHz) δ (ppm); 38.5 (C-1), 21.1 (C-2), 80.7 (C-3), 38.2 (C-4), 55.5 (C-5), 18.4 (C-6), 34.4 (C-7), 40.9 (C-8), 50.5 (C-9), 37.2 (C-10), 21.1 (C-11), 23.9 (C-12), 36.3 (C-13), 42.9 (C-14) 25.3 (C-15), 35.7 (C-16), 43.1 (C-17), 48.4 (C-18), 48.2 (C-19), 151.1 (C-20), 29.9 (C-21), 40.1 (C-22), 27.6 (C-23), 16.7 (C-24), 16.3 (C-25), 16.1 (C-26), 14.7 (C-27), 18.1 (C-28), 109.5 (C-29), 19.4 (C-30), 173.8 (C-1′), 28.1 (C-2′).

*Cis*-*p*-*coumaric acid* (**2**). C_9_H_8_O_3_ white amorphous powder (10 mg) ^1^H-NMR (CDCl_3_, 400 MHz): δ (ppm); 7.63 (d, *J* = 8.8 Hz, H-2, 6), 6.83 (d, *J* = 12.5 Hz, H-7), 6.79 (d, *J* = 8.8, H-3, 5), 5.82 (d, *J* = 12.5 Hz, H-8). ^13^C-NMR (CDCl_3_, 100 MHz) δ (ppm); 127.6 (C-1), 132.5 (C-2, 6), 115.1 (C-3, 5), 156.9 (C-4), 143.4 (C-7), 117.4 (C-8), 166.9 (C-9).

*Lupeol* (**3**). C_30_H_50_O crystals (45 mg) ^1^H-NMR (CDCl_3_, 400 MHz): δ (ppm); 4.68, 4.56 (2H, s, H-29a, 29b), 3.18 (1H, dd, *J* = 4.76 Hz, 11.2 Hz, H-3), 0.75, 0.78, 0.82, 0.94, 0.96, 1.02, 1.25 (each 3H, s, CH_3_ x 7). ^13^C-NMR (CDCl_3_, 100 MHz) δ (ppm); 38.2 (C-1), 25.3 (C-2), 79.2 (C-3), 38.7 (C-4), 55.4 (C-5), 18.5 (C-6), 34.4 (C-7), 40.9 (C-8), 50.6 (C-9), 37.3 (C-10), 21.1 (C-11), 27.6 (C-12), 39.0 (C-13), 42.9 (C-14) 27.6 (C-15), 35.7 (C-16), 42.9 (C-17), 48.5 (C-18), 48.1 (C-19), 151.1 (C-20), 29.9 (C-21), 40.2 (C-22), 28.1 (C-23), 15.5 (C-24), 16.3 (C-25), 16.2 (C-26), 14.7 (C-27), 18.5 (C-28), 109.5 (C-29), 19.5 (C-30).

*β-Sitosterol* (**4**). C_29_H_50_O crystals (20 mg) ^1^H-NMR (CDCl_3_, 400 MHz): δ (ppm); 3.52 (1H, m), 5.34 (1H, d), 0.92 (3H, d), 0.84 (3H, t), 0.82 (3H, d), 0.80 (3H, s), 0.67 (3H, s), 1.00 (3H, s). ^13^C-NMR (CDCl_3_, 100 MHz) δ (ppm); 37.4 (C-1), 31.8 (C-2), 71.9 (C-3), 42.5 (C-4), 140.9 (C-5), 121.9 (C-6), 32.1 (C-7), 32.1 (C-8), 50.3 (C-9), 36.7 (C-10), 21.2 (C-11), 39.9 (C-12), 42.5 (C-13), 56.9 (C-14) 26.2 (C-15), 28.4 (C-16), 56.2 (C-17), 36.3 (C-18), 19.2 (C-19), 34.1 (C-20), 26.2 (C-21), 45.9 (C-22), 23.2 (C-23), 12.1 (C-24), 29.3 (C-25), 19.9 (C-26), 19.5 (C-27), 18.9 (C-28), 12.0 (C-29).

*Trans*-*p*-*coumaric acid* (**5**). C_9_H_8_O_3_ white amorphous powder (10 mg) ^1^H-NMR (CDCl_3_, 400 MHz): δ (ppm); 7.64 (d, *J* = 16 Hz, H-7), 7.42 (d, *J* = 8.8 Hz, H-2,6), 6.84 (d, *J* = 8.8 Hz, H-3,5), 6.29 (d, *J* = 16 Hz, H-8) ^13^C-NMR (CDCl_3_, 100 MHz) δ (ppm); 167.8 (C-9), 115.9 (C-8), 144.4 (C-7), 130.1 (C-2, 6), 116.0 (C-3, 5), 157.8 (C-4), 127.4 (C-1).

*Linoleic acid* (**6**). C_18_H_30_O_2_ Yellow oil (34 mg) EIMS *m/z* 280 [M]^+^ (17), 262 (2), 237 (1), 209 (2), 182 (3), 166 (1), 150 (7), 136 (10), 123 (15), 109 (34), 95 (70), 81 (93), 67 (100), 55 (2), 45 (5).

*(+)-Catechin* (**7**). C_15_H_14_O_6_ powder green amorphous (13 mg) ^1^H-NMR (CDCl_3_, 400 MHz): δ (ppm): 2.52 (dd, *J* = 8 Hz), 2.87 (dd, *J* = 5.6 Hz), 3.97 (m), 4.56 (d, *J* = 7.2 Hz), 5.85 (d, *J* = 2.4 Hz), 5.93 (d, *J* = 2.4 Hz), 6.72 (dd, *J* = 8.4 Hz, *J* = 2 Hz), 6.76 (d, *J* = 8.4 Hz), 6.83 (d, *J* = 2 Hz); ^13^C-NMR (CDCl_3_, 100 MHz) δ (ppm); 132.2 (C-1′), 82.9 (C-2), 115.3 (C-2′), 68.8 (C-3), 146.2 (C-3′-4′), 28.5 (C-4), 157.6 (C-5), 116.1 (C-5′), 96.3 (C-6), 120.0 (C-6′), 157.8 (C-7), 95.5 (C-8), 156.9 (C-9), 100.8 (C-10). ${\left[\alpha \right]}_{D}^{27}$ = +3.6 (c 0.3, MeOH).

*Afzelin* (**8**). C_21_H_20_O_10_ powder (13 mg) ^1^H-NMR (CDCl_3_, 400 MHz): δ (ppm): 7.77 (d, *J* = 8.8 Hz), 6.93 (d, *J* = 8.8 Hz), 6.38 (d, *J* = 2.4 Hz), 6.20 (d, *J* = 2 Hz), 5.38 (d, *J* = 1.6 Hz), 4.22 (*J* = 1.6 Hz), 3.71 (m), 3.59 (s), 3.34 (m), 0.92 (d); ^13^C-NMR (CDCl_3_, 100 MHz) δ (ppm); 122.6 (C-1′), 103.5 (C-1″), 158.6 (C-2), 131.9 (C-2′, 6′), 72.1 (C-2’), 136.2 (C-3), 116.5 (C-3′, 5′), 72.0 (C-3″), 179.6 (C-4), 161.6 (C-4′), 73.2 (C-4″), 163.2 (C-5), 71.9 (C-5″), 99.8 (C-6), 17.7 (C-6″), 165.9 (C-7), 94.8 (C-8), 159.3 (C-9), 105.9 (C-10).

*Quercitrin* (**9**). C_21_H_20_O_11_ amorphous yellow powder (15 mg) ^1^H-NMR (CDCl_3_, 400 MHz): δ (ppm): 7.33 (d, *J* = 2 Hz), 7.31 (dd, *J* = 2, 8 Hz), 6.91 (d, *J* = 8.4 Hz), 6.37 (d, *J* = 2.4 Hz), 6.20 (d, *J* = 1.6 Hz), 5.35 (d, *J* = 1.6 Hz), 4.22 (dd, *J* = 2, 3.6 Hz), 3.75 (dd, *J* = 3.6, 9.2 Hz), 3.42 (m), 3.35, 0.94 (d, *J* = 6 Hz); ^13^C-NMR (CDCl_3_, 100 MHz) δ (ppm); 122.9 (C-1′), 103.6 (C-1″), 158.5 (C-2), 116.4 (C-2′), 72.0 (C-2′), 136.2 (C-3), 146.4 (C-3′), 72.1 (C-3″), 179.7 (C-4), 149.8 (C-4′), 73.3 (C-4″), 163.2 (C-5), 116.9 (C-5′), 71.9 (C-5″), 99.8 (C-6), 122.9 (C-6′), 165.9 (C-7), 94.7 (C-8), 159.3 (C-9), 105.9 (C-10).

## Figures and Tables

**Table 1:** In vitro anti α-amylase and α-glucosidase activity (as IC_50_ values) of extracts and compounds isolated from *Muehlenbeckia tamnifolia*.

No.	Extract/Compound	IC_50_ (μg/mL, μM *)	
		α-amylase	α-glucosidase
**A**	Hexane extract	625	48.22
**B**	EtOAc extract	-	416.67
**C**	MeOH extract	-	19.22
**1**	Lupeol Acetate	-	-
**2**	*cis*-*p*-coumaric	-	-
**3**	Lupeol	-	-
**4**	β-sitosterol	-	-
**5**	*trans-p*-coumaric	-	-
**6**	Linoleic *	-	0.42
**7**	(+)-Catechin *	-	5.50
**8**	Afzelin *	-	3.56
**9**	Quercitrin *	-	7.77
**D**	Acarbose	10	377

Note: * Max concentration tested: 10 mg/mL for extracts and 1 mM for compounds.
